# Prokaryotic Population Dynamics and Viral Predation in a Marine Succession Experiment Using Metagenomics

**DOI:** 10.3389/fmicb.2019.02926

**Published:** 2019-12-19

**Authors:** Jose M. Haro-Moreno, Francisco Rodriguez-Valera, Mario López-Pérez

**Affiliations:** ^1^Evolutionary Genomics Group, División de Microbiología, Universidad Miguel Hernández, Alicante, Spain; ^2^Laboratory for Theoretical and Computer Research on Biological Macromolecules and Genomes, Moscow Institute of Physics and Technology, Dolgoprudny, Russia

**Keywords:** metagenomics, rare biosphere, metagenome-assembled genomes, Mediterranean Sea, host viral interaction, metagenomic recruitment, bottle effect, microbial diversity

## Abstract

We performed an incubation experiment of seawater confined in plastic bottles with samples collected at three depths (15, 60, and 90 m) after retrieval from a single offshore location in the Mediterranean Sea, from a late summer stratified water column. Two samples representative of each depth were collected and stored in opaque bottles after two periods of 7 h. We took advantage of the “bottle effect” to investigate changes in the natural microbial communities (abundant and rare). We recovered 94 metagenome-assembled genomes (MAGs) and 1089 metagenomic viral contigs and examined their abundance using metagenomic recruitment. We detected a significant fast growth of copiotrophic bacteria such as *Alteromonas* or *Erythrobacter* throughout the entire water column with different dynamics that we assign to “clonal,” “polyclonal,” or “multispecies” depending on the recruitment pattern. Results also showed a marked ecotype succession in the phototropic picocyanobacteria that were able to grow at all the depths in the absence of light, highlighting the importance of their mixotrophic potential. In addition, “wall-chain-reaction” hypothesis based on the study of phage–host dynamics showed the higher impact of viral predation on archaea in deeper waters, evidencing their prominent role during incubations. Our results provide a step forward in understanding the mechanisms underlying dynamic patterns and ecology of the marine microbiome and the importance of processing the samples immediately after collection to avoid changes in the community structure.

## Introduction

Meta-omic and single-cell genomic approaches have opened a new window to a vast world of aquatic microbes known before only through their rRNA sequences, increasing the level of resolution to a much finer and informative level. The relative easiness of sampling and size fractionation by different filter sizes have been essential to facilitate the analysis, and large datasets have been generated. Large-scale marine metagenomic studies (Sunagawa et al., 2015; Biller et al., 2018a) have aided in the discovery of an astonishing diversity of the prokaryotic community. However, most of our knowledge is frequently focused on abundant organisms, and much less attention has been devoted to low-abundance populations. This part of the microbial diversity, also called the “rare biosphere” (Sogin et al., 2006), has an arbitrarily defined relative abundance < 0.1% of the total community (Sogin et al., 2006). Nevertheless, these underrepresented populations might eventually become dominant in response to particular environmental disturbances helping community resilience (Jones and Lennon, 2010). In addition, they can also serve as an important reservoir of genetic and functional diversity (Lynch and Neufeld, 2015). Recently, these populations have been designated as “conditionally rare taxa” (Shade et al., 2014) and although the ecological roles of these rare microorganisms remain unclear, a conceptual model based on their dynamics has been proposed in order to improve our understanding about the microbial community stability and resilience in the ecosystem (Shade and Gilbert, 2015).

One typical example of a change in the environment is the deep winter convection within the water column in temperate waters that brings nutrients to surface waters and produce phytoplankton blooms. This spring bloom is followed by changes in the composition of bacterial and archaeal communities due to their decay products (Fuhrman et al., 2015). One recent study examined fine-scale variations in the marine microbiome in the Mediterranean Sea based on metagenomic assembly and recruitment showed that during the winter mixing period only some groups of bloomers (*r*-strategists) characterized by having a large estimated genome size and a high GC content were favored (Haro-Moreno et al., 2018). These bloomers have been defined as microbes that exhibit fast growth under conditions of a sudden increase in the availability of resources in the environment and are the main contributors of rare taxa (Eilers et al., 2000). This dynamic of temporary changes in the population of some microbes in response to both biotic and abiotic factors can occur on a time scale of hours or even months (Fuhrman et al., 2015; Bunse and Pinhassi, 2017). It is clear that seasonal cycles are complex, but with the data collected from different time-series studies (Bunse and Pinhassi, 2017), it has been demonstrated that the distribution and abundance of microorganisms were highly predictable and determined by seasonal factors (Fuhrman et al., 2006). Their frequency suggests that they fulfill an essential ecological function in the environment.

Microbial succession experiments using seawater confinement are often used to characterize the influence or responses to naturally occurring phytoplankton blooms and their subsequent demise, nutrient fluxes, dissolved organic matter, or viral infection and lysis (Riemann et al., 2000; Schäfer et al., 2000; Carlson et al., 2002; Pinhassi et al., 2004; Allers et al., 2007; McCarren et al., 2010; Fuhrman et al., 2015; Hou et al., 2018). During this artificial confinement, bacteria can grow up ten to thousands of times (Waksman and Carey, 1935), and the natural community can be altered by the growth of opportunistic bacteria (Ferguson et al., 1984; Lee and Fuhrman, 1991), it is the so-called “bottle effect” (Waksman and Carey, 1935; Zobell and Anderson, 1936). Within a closed system, the nutrient balance will be negative and, in the end, only a few species will be able to survive after a few days without reaching equilibrium. This variation leads to a decrease of the total microbial diversity in the sample (Massana et al., 2001), and has profound implications for studies that imply long incubation times such as those analyzing the degradation of dissolved carbon and microbial respiration in aquatic habitats (Baltar et al., 2012) or the denitrification rate in soil samples (Hartzog et al., 2017). Previous studies suggest that this effect favors heterotrophic over autotrophic bacteria (Calvo-Díaz et al., 2011) and mainly the growth of Gammaproteobacteria (Eilers et al., 2000; Stewart et al., 2012; Dinasquet et al., 2013). These effects are probably due to the combination of several factors such as biofilm formation, temperature, or binding to particles on the surface of the container (Fogg and Calvario-Martinez, 1989; Eilers et al., 2000).

It has been shown that within the photic zone of thermally stratified water columns, there are at least three clearly different regions with significant differences in the microbial composition, the deep chlorophyll maximum (DCM), the upper photic (UP) located above, and lower photic (LP) below the DCM (Haro-Moreno et al., 2018). In order to obtain more insights into the dynamics of microbial communities in these three layers of the water column, we combined culture-based incubations with metagenomic assembly and recruitment. We performed a mesocosm experiment of seawater confined in plastic bottles after retrieval from a single offshore location in the Mediterranean taking advantage of this “bottle effect.” One sample representative of each depth was analyzed after 7 and 14 h. Although limited to a single location in the Mediterranean Sea, these results not only improve our understanding of the dynamic patterns, ecology, and taxonomy of the marine microbiome but also support the previous suggestions about the importance of to maintain a high intra-population diversity in the more abundant microbes and the presence of bloomers as ecological drivers of environmental perturbations (Shade and Gilbert, 2015; Jousset et al., 2017; Jia et al., 2018).

## Materials and Methods

### Sampling Collection and Processing

Samples from three different depths (15, 60, and 90 m) were collected on 15^th^ October 2015 at a single site from the Western Mediterranean (37.35361°N, 0.286194°W), at approximately 60 nautical miles off the coast of Alicante, Spain, from the research vessel “García del Cid.” For each depth, one seawater sample (200 L each) was collected and quickly filtered as described in Haro-Moreno et al. (2018). These samples corresponded with 0 h incubation time. Two more samples per depth (six in total) were collected and stored in 30 L opaque bottles at air temperature (ca. 25°C), which were previously washed with NaOH 0.1 M. Bottles were rinsed three times with seawater from the same depth. Three samples were filtered at 7 h post-incubation and two after 14 h.

Independently of the time of the incubation, all seawater samples were sequentially filtered on board through 20, 5, and 0.22 μm pore size polycarbonate filters (Millipore). Filters were immediately frozen on dry ice and stored at −80°C until processing. DNA was extracted from the 0.22 μm filter as previously described (Martin-Cuadrado et al., 2008), and sequenced using Illumina Hiseq-4000 (150 bp, paired-end read) (Macrogen, Republic of Korea). Unfortunately, 14 h sample from 90 m did not pass quality control and therefore it was discarded for sequencing.

### Assembly of Metagenomic Reads and Contig Annotation

Individual metagenomic reads were trimmed with Trimmomatic (Bolger et al., 2014) and assembled with IDBA-UD (Peng et al., 2012). ORFs from the assembled contigs were predicted using Prodigal (Hyatt et al., 2010). tRNAscan-SE (Lowe and Eddy, 1996), ssu-align (Nawrocki, 2009), and meta-rna (Huang et al., 2009) were used to predict the tRNA and rRNA genes. For taxonomic and functional annotation, predicted protein sequences were compared against NCBI NR database using DIAMOND (Buchfink et al., 2015), and against COG (Tatusov et al., 2001) and TIGRFAM (Haft et al., 2001) using HMMscan (Eddy, 2011).

### 16S rRNA Classification

Using USEARCH6 (Edgar, 2010), a database containing non-redundant 16S rRNA sequences downloaded from the RDP database (Cole et al., 2014) was used to identify candidate 16S rRNA gene sequences in the raw metagenomes. Only candidate reads that matched this database with an E-value < 10^–5^ were considered, and later aligned to archaeal, bacterial, and eukaryal 16S/18S rRNA HMM models using ssu-align (Eddy, 1995) to identify true sequences and to remove possible mismatches and/or putative 18S rRNA sequences. Only hits to 16S rRNA sequences were then classified using USEARCH6 against the RDP database and classified into a high-level taxon if the sequence identity was ≥80% and the alignment length ≥90 bp. Sequences failing these thresholds were discarded.

### GC Content and Cross-Comparison of the Metagenomic Reads

GC content was calculated using the gecee program from the EMBOSS package (Rice et al., 2000). A cross-comparison among metagenomic samples using the curated 16S rRNA reads (see section “16S rRNA Classification”) was performed using Simka (Benoit et al., 2016) with *k* = 21, which represents 95% identity. Samples were then clustered using the abundance-based Bray–Curtis dissimilarity distance (Benoit et al., 2016).

### Binning and Genome Reconstruction

Only assembled contigs longer than 5 kb were used for the recovery of novel organisms. Taxonomic affiliation, tetranucleotide frequencies, GC content, and coverage values within the metagenomes collected in this work and in Haro-Moreno et al. (2018) were used to bin the contigs into metagenome-assembled genomes (MAGs). Tetranucleotide frequencies were computed using wordfreq program in the EMBOSS package. Contigs were taxonomically classified to high-level taxon if >50% of the genes shared the same taxonomy. In order to improve the completeness and remove the redundancy, MAGs recovered from the different samples that shared an average nucleotide identity (ANI) > 99.5% were combined. Using BWA (Li and Durbin, 2009), pooled contigs were then used to retrieve the short-paired reads that mapped onto the contigs. These reads were then pooled and assembled together with the contigs using SPAdes (Bankevich et al., 2012). MAG completeness was estimated by detecting the percentage of single-copy genes coded within MAGs after comparison against two different universal gene sets (Raes et al., 2007; Albertsen et al., 2013) using HMMscan, and by CheckM (Parks et al., 2015). CheckM also provided the degree of contamination. Only MAGs with a completeness in any of the three methods used > 50% and a contamination <5% were kept for further analyses.

### Metagenomic Read Recruitments

Genomes of known marine microbes together with several MAGs and SAGs recovered from several studies (Ghai et al., 2013; Mizuno et al., 2015; Haro-Moreno et al., 2017, 2018; Parks et al., 2017; Tully et al., 2018), including this work, were used to recruit reads from our metagenomic (NCBI BioProjects PRJNA257723 and PRJNA352798) (López-Pérez et al., 2017; Haro-Moreno et al., 2018) and *Tara* Mediterranean (ENA PRJEB1787) (Sunagawa et al., 2015) datasets using BLASTN (Altschul et al., 1997) (99% identity, > 50 bp alignment). Genomes that recruited less than three reads per kilobase of genome per gigabase of metagenome (RPKG) were not considered.

### Phylogenomic Classification of the Reconstructed MAGs

We performed a phylogenomic analysis using PhyloPhlAn (Segata et al., 2013) to classify the reconstructed genomes, together with all the reference genomes that recruited in any of the samples. A total of 263 proteins were shared in all the genomes.

### Functional Classification and Analysis of the Assembled Proteins

To infer the potential metabolic function of the assembled prokaryotic community that grew during the experiment, only contigs longer than 1 kb which increased their relative abundance (measured in RPKG) more than three times and recruited more than 3 RPKG after 14 h post-confinement were selected. Proteins encoded in these contigs were compared against the SEED subsystems (Overbeek et al., 2005) databases using DIAMOND (blastp option, top hit, ≥50% identity, ≥50% alignment length, E-value < 10^–5^) and against the CAZy database (Lombard et al., 2014) using HMMscan (E-value < 1e^–8^).

### Viral Contigs and Host Prediction

In order to identify the viral origin of the contigs larger than 10 kb, we performed a manual inspection based on the resemblance to known phages similar to methods that have been previously described (Mizuno et al., 2013; López-Pérez et al., 2017). These sequences were also filtered using VirFinder (Ren et al., 2017). Several host prediction approaches have been used such as tRNA matches, CRISPR spacers, presence of Auxiliary Metabolic Genes (AMGs), all-versus-all comparisons, and terminase phylogeny (Mizuno et al., 2013).

### Data Availability

Metagenomic datasets have been submitted to NCBI SRA and are available under BioProject accession numbers PRJNA352798 (Med-OCT2015-15m [SRR5007106], Med-OCT2015-60m [SRR5007118], Med-OCT2015-90m [SRR5007139], Med-OCT2015-15m7h [SAMN10839273], Med-OCT2015-15m14h [SAMN10839292], Med-OCT2015-60m7h [SAMN10839294], Med-OCT2015-60m14h [SAMN10839296], and Med-OCT2015-90m7h [SAMN10839330]). The reconstructed genomes have been deposited as BioSample SAMN10841217 to SAMN10841310 under BioProject PRJNA352798.

## Results and Discussion

In order to investigate the changes in the microbial community during an incubation (“bottle effect”) experiment, samples were collected at three different depths [15 m (UP); 60 m (DCM); and 90 m (LP)] of the stratified water column in an off-shore Western Mediterranean sampling site described previously (Haro-Moreno et al., 2018). After 7 and 14 h, the bottle content was swiftly filtered on board through 20, 5, and 0.22 μm pore size filters. Metadata and sequencing results are described in Supplementary Table S1.

First, we analyzed the relationship of the different time-confined samples compared with their corresponding metagenomes of the pre-confinement filter (those directly filtered from the hose bringing the water from the corresponding depth) (Haro-Moreno et al., 2018) using the number of reads that matched to the 16S rRNA gene metagenomic fragments. These data were then used to perform a cladogram based on a dissimilarity pairwise comparison among the samples (Supplementary Figure S1). We included the samples from 30 and 75 m deep collected on the same day and others from different years and depths from the same location, as well as two more samples collected during the winter when the photic water column was mixed (Haro-Moreno et al., 2018), as references for the clustering. These samples were previously used to analyze the fine-scale variations in the water column microbiome. Clustering showed that incubation samples at 15 m remained more similar to the original sample than the deeper samples and separate, for example, from the 30 m samples that clustered together within the UP region. These small differences could be due to: (i) low microbial diversity observed in the 15 m sample in comparison to the rest of the water column (Haro-Moreno et al., 2018) and (ii) the effect of temperature, since the temperature at which the bottles were kept (ca. 25°C) was more similar to that of the sea surface (22.9°C), while deeper samples showed lower temperature values (14.5 and 13.8°C, respectively). More marked were the differences in deeper waters where the confined sample at 90 m (7 h) clustered together with the group formed by 75 and 90 m samples (Supplementary Figure S1). At the DCM, while the 7 h sample was similar to the DCM reference, the sample after 14 h was slightly distant from both samples. In another branch close to the DCM samples, we found the mixed water column (winter) samples.

In the same way, analysis of the prokaryotic community structure at the level of phylum derived from 16S rRNA gene metagenomic fragments revealed a shift in microbiome composition during the confinement (Supplementary Figure S2). We found that at the DCM Archaea represented nearly 11% of the total population but decreased to approximately 2% in only 14 h (Supplementary Figure S2). Specifically, at the DCM sample, Poseidonarchaea and Thalassoarchaea (formerly Marine Group II Euryarchaeota) (Rinke et al., 2019) decreased from ca. 5% of the population in the time 0 h sample to close to nothing in the 14 h sample (at 7 h their decrease was already very apparent) (Supplementary Figure S2). In deeper waters (90 m), while MGII/III Euryarchaeota also decreased, Thaumarchaeota increased slightly after 7 h conditions. Whereas the proportion of Bacteroidetes (mainly *Flavobacteriaceae*) and Gammaproteobacteria remained constant during surface seawater confinement, both increased with time in samples from deeper layers of the photic zone. The most dominant group in all depths, Alphaproteobacteria, had a different behavior, decreasing with time at 15 and 90 m and increasing in the DCM. Noteworthy was the increase of Cyanobacteria regardless of the depth, although all the samples were incubated in opaque bottles (Supplementary Figure S2). The proportion of 16S rRNA reads assigned to unclassified bacteria decreased with time in all depths, likely due to the increase of known microbes, probably bloomers that are easy to obtain in pure culture.

Analysis of the GC content from metagenomic reads showed a trend, independently of the depth, toward a GC of *ca*. 41% 14 h post-confinement (Supplementary Table S1), value previously found for the winter samples (Haro-Moreno et al., 2018). It has been suggested that these changes in genomic features are evolutionary strategies in response to environmental conditions such as the availability of nitrogen and energy (Mende et al., 2017).

### MAGs Recovery and Phylogeny

**FIGURE 1:**
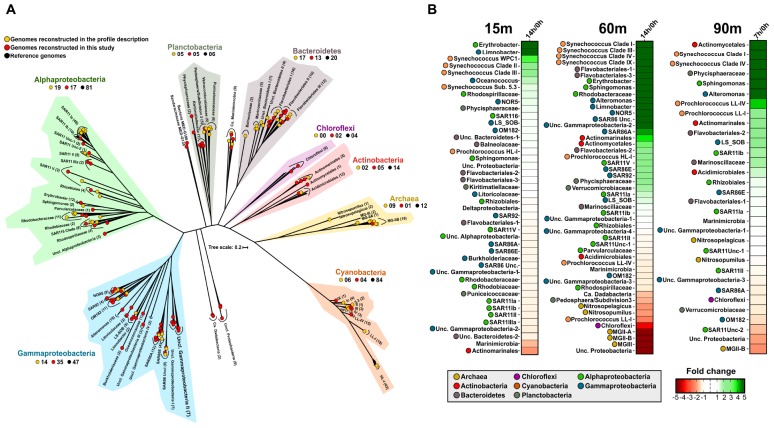
**(A)** Phylogenetic analysis of the 445 genomes that recruit in any of the samples. A maximum likelihood genome tree was constructed with 100 bootstraps using 262 conserved proteins. Branches in the tree are sorted and colored according to their taxonomy. Metagenome-assembled genomes (MAGs) reconstructed in this study are indicated as red circles and those recovered in a previous study (Haro-Moreno et al., 2018) in yellow. Black circles show reference genomes. **(B)** Metagenomic recruitment (≥ 99% identity) of genomes that recruited at least three RPKGs in any of the samples. Genomes are sorted with colored dot according to their taxonomy.

From the stored bottles, we were able to recover 94 novel (< 99% ANI) MAGs, all of them with an estimated completeness higher than 50% and less than 5% contamination (Supplementary Table S2). In order to cover as much as possible the diversity of these samples, we used the MAGs together with those recovered from the same and other locations (Ghai et al., 2013; Mizuno et al., 2015; Haro-Moreno et al., 2017, 2018; Parks et al., 2017; Tully et al., 2018), several single-cell genomes (Berube et al., 2018), and marine reference culture genomes (adding up to more than 10,000 individual genomes in total). After de-replication at 99% identity, we selected only genomes that recruited at least three RPKG with an identity higher than 99%. In the end, only 445 genomes recruited with this threshold in at least one of the samples (Supplementary Table S3) and we focused on them. Phylogenomic analysis using shared proteins (262) among the recruiting genomes placed all of them consistently with the same taxonomic groups found by 16S rRNA metagenomic fragment analysis (Figure 1A and Supplementary Figure S2). A total of 219 genomes (∼50%) were classified within the phylum Proteobacteria that was the major contributor among them, consistent with the high numbers of 16S rRNA reads that were classified into this group (Figure 1A and Supplementary Figure S2). Representatives of Gammaproteobacteria were the most abundant, followed by Alphaproteobacteria, Bacteroidetes, Planctobacteria, and Actinobacteria (Figure 1A). Although it has been possible to recover almost 100 new high-quality genomes through assembly, it is known that very frequently the assembly has biases on genomes that are not very abundant or with great genomic diversity. For this reason, the recruitment of genomes from reference microbes obtained from other studies is an advantage in order to bring to light all the diversity hidden behind a sample.

### MAGs Abundance in Marine Metagenomes

However, these changes in the prokaryotic community should be interpreted with caution since this is a semi-quantitative study and there are stochastic effects over which we have no control, e.g., interactions between phytoplankton and associated bacteria (Amin et al., 2015). For this reason, we sought to examine the relative abundance of all the MAGs across these incubation samples and other samples collected from the Mediterranean Sea, including samples from the same location in different years and depths (Haro-Moreno et al., 2017; López-Pérez et al., 2017) as well as from *Tara* Oceans database (Sunagawa et al., 2015). We performed fragment recruitment analysis of all these MAGs that recruited in at least two samples with a similarity ≥ 95%. The resulting heatmap based on the clustering of the samples revealed five main branches, three of them corresponding to the previously defined regions (UP, DCM, and LP) (Haro-Moreno et al., 2018), the bathypelagic sample (1000 m) that could be considered as an outgroup compared to all the other samples and another branch where is included the 60m-14h sample (Supplementary Figure S3). All the samples appeared associated with the corresponding partners according to their depth. However, while the initial times clustered with similar samples at both 15 and 60 m, the incubation samples appeared as outgroups indicating a slight change in the community. The exception was the 60m-14h sample that formed a different branch with three samples from the Eastern Mediterranean (Aegean and Ionian Sea). These samples clustered together, probably due to the ultraoligotrophic conditions of this region (Moutin et al., 2012). The lack of metagenomes from LP regions limits the assessment of incubations on these samples but, although they appeared in the same branch, sample from 90 m was more similar to the 75 m than to the 90m–7h. Together, these results showed a shift in microbiome composition during the confinement (Supplementary Figure S3), much more accentuated in the DCM. Thus, the composition of the prokaryotic community in the incubation samples could be used to detect the “bottle effect” in other samples as quality control. These results highlight the DCM not only as the section of the water column with the highest population density of marine microbes but also the reservoir of greatest diversity. Furthermore, it is evident that maintaining incubated seawater without adding any nutrients, but also to a lesser extent, light, oxygen availability, and water temperature change the structure of the microbial community in a short time.

These data also have important implications in the implementation and development of experimental systems in marine microbial ecology. Very often, samples in oceanic expeditions are retrieved and stored in plastic bottles or other kinds of containers while the large volume of water involved is pumped through the filters. For example, when deep samples are taken, the Niskin bottle takes several hours to come from the deep (more than 1 h/1000 m). The use of this short−term temporal dynamics experiment revealed that confinement periods can produce changes in the overall community structure in 7 h (probably less), although changes keep happening after 14 h without adding any nutrient. Our results highlight the importance of processing the samples swiftly after collection, to avoid changes and obtain the most accurate representation of the *in situ* community.

### Microbial Succession During the “Bottle Effect”

These metagenomics approaches allowed us not only to recover nearly a hundred new genomes but also obtain a finer resolution at the species or ecotype level, unlike 16S rRNA-related techniques. We have first analyzed those groups where the most significant changes in relative abundance occur during incubation.

#### Bloomers or *r*-Strategists

Metagenomic recruitments showed a pronounced increase in some marine copiotrophic bacteria (Figure 1B). For instance, members of *Erythrobacter* and *Limnobacter* (recently classified within the class Gammaproteobacteria; Parks et al., 2018) rose after confinement of the 15 and 60 m depth samples (Figure 1B). At 15 m, we also observed the increase of the MAG Oceanococcus MED-G154 (Gammaproteobacteria) and the MAG Sandaracinaceae MED-G138 (Deltaproteobacteria). However, they disappeared in the 14 h sample. *Alteromonas* also increased in our experiment, but only in the DCM and LP post-confinement samples. Genomes of *Sphingomonas* increased at all depths. This genus was only found *in situ* at 15 and 1000 m (Haro-Moreno et al., 2018), so their growth in the DCM and LP regions, on which they were not detected before confinement, strengthens the previously advanced idea that *Sphingomonas* behaves as a truly eurybathic microbe (Haro-Moreno et al., 2018). All these microorganisms are considered opportunistic (bloomers) with trends toward large genome size and encoding a wide variety of metabolic pathways to exploit a wide range of substrates. Results suggest the shift of the initial community toward organisms that can be easily retrieved in pure culture (*r*-strategists), as it has been previously described (Eilers et al., 2000).

**FIGURE 2:**
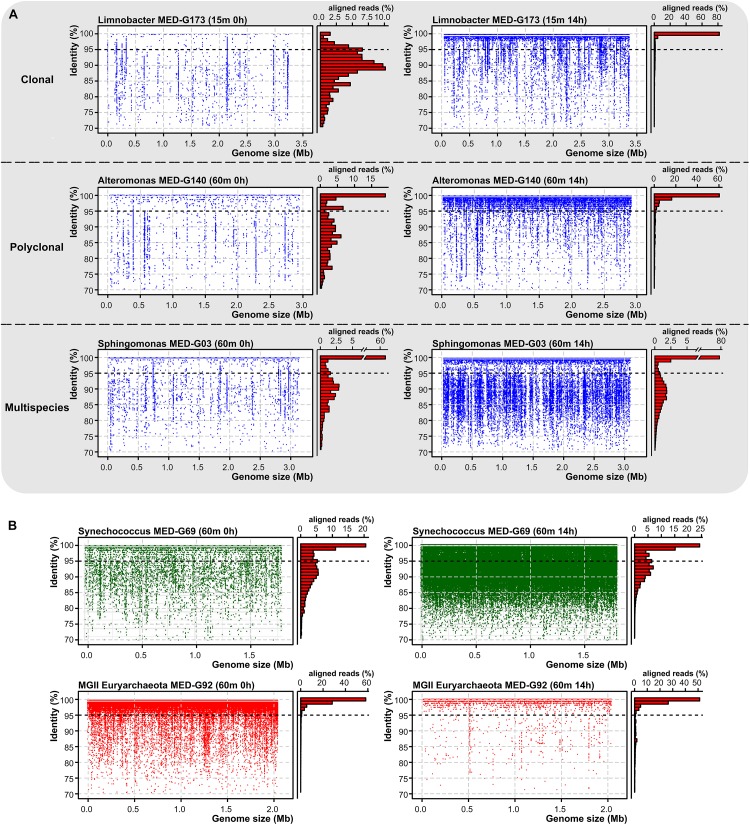
**(A)** Recruitment plots of three marine species with distinct behaviors, *Limnobacter* MED-G173 (clonal), *Alteromonas* MED-G140 (polyclonal), and *Sphingomonas* MED-G03 (multispecies) at time 0 and 14 h post-confinement. On the left, each blue dot represents a metagenomic read. Histogram on the right shows the relative percentage of aligned reads in intervals of 1% identity. Black dashed line indicates the species threshold (95%). **(B)** Recruitment plot of two metagenome-assembled genomes at the DCM, highlighting the differences in abundance levels across time.

The recruitment plots in Figure 2A show that although these genomes recruit along their entire lengths, they showed different genome-level diversity patterns during the bottle confinement. In the case of *Limnobacter*, we observed a likely clonal amplification, with a linear recruitment plot with most reads having >99% identity to the reference genome. However, *Alteromonas* and *Erythrobacter* showed a “polyclonal” amplification with a similarity cloud down to a nucleotide identity of 95% (Figure 2A). In the case of *Sphingomonas*, recruitment indicated that several species of the genus (ANI below 95%) were capable of growing at the same time representing a “multispecies” amplification (Figure 2A).

#### Picocyanobacteria

Unexpectedly, we found a very significant increase in the number of reads assigned to picocyanobacteria, mainly *Synechococcus*, even at depths (60 and 90 m deep) (Figure 2B) where they were not previously detected (Haro-Moreno et al., 2018). It is known that picocyanobacteria are capable of mixotrophic growth when they are in darkness, probably due to their interaction with heterotrophs as has been previously suggested (Yelton et al., 2016), and particularly *Synechococcus* contains several organic carbon transporters in their relatively large genomes. However, different ecotypes (Scanlan, 2012) were found to increase differentially depending on the depth of the sample. In the 15 m sample, members of the clades II, III (exclusive of the Mediterranean Sea; Farrant et al., 2016) and WPC1 (all of the subcluster 5.1), and members of the subcluster 5.3 increased their recruitment. Members of these groups are found primarily in warm, coastal, and off-shore areas (Scanlan, 2012; Farrant et al., 2016). In the DCM sample, members of the clades I, III, IV, and IX were found to increase their abundance (Figure 1B). At the 90 m sample, only clades I and IV were found to increase after confinement although they were nearly absent in the sample processed directly. Members of the clade I had been characterized as opportunists in cold/coastal habitats (Scanlan, 2012), reaching higher numbers after environmental perturbation (Dufresne et al., 2008). Therefore, it is plausible that at least clade I experienced an increase in a short time. Remarkably, in the absence of significant light, only one picocyanobacterial ecotype was found to decrease at the DCM depth sample belonging to the Low-Light I of *Prochlorococcus*, although both Low-Light I and IV increased their abundance at the confined 90 m sample.

The presence of fast growers like *Erythrobacter* at 15 m or *Alteromonas* at 60 and 90 m might enhance the growth of the phototrophs (both *Synechococcus* and *Prochlorococcus*), as previously described (Sher et al., 2011; Christie-Oleza et al., 2017; Biller et al., 2018b). They may remove certain toxic products (Morris et al., 2011) or provide energy in the form of certain organic reduced compounds, which are then oxidized via the oxidative pentose (Stal and Moezelaar, 1997) or the Entner–Doudoroff pathways, the latter proposed to be the main route of glucose degradation during mixotrophic conditions (Chen et al., 2016). The increase of picocyanobacteria in our samples is consistent with the results that shows the stability of these autotrophic bacteria during a short period of darkness (*ca*. 14 h) (Biller et al., 2018b). However, our experiment represented a more complex community that a single co-culture of *Prochlorococcus*-*Alteromonas* or *Synechococcus*-*Roseobacter*, and other abiotic and biotic factors might play a role in the growth of the phototrophs in the dark.

Remarkable is that part of the great diversity hidden until now seems to be encoded within large groups such as marine picocyanobacteria, mainly *Synechococcus*. Results clearly showed a succession of different ecotypes with a different role within the ecosystem. Unlike all the heterotrophs obtained (*Alteromonas*, *Sphingomonas*, or *Erythrobacter*), whose metagenomic recruitment showed a low genomic diversity, within these major clades, several ecotypes coexist simultaneously overcoming the disturbances of the environment. Therefore, those ecotypes that are not dominant in these conditions may be dominant in others where biotic or abiotic factors are propitious for their growth consigning the other ecotypes to part of the “rare biosphere.”

### Changes in the Dominant Bacterial Taxa

One of the factors usually overlooked in such studies (Massana et al., 2001; Lynch and Neufeld, 2015; Jia et al., 2018) is how the growth of these microbes affects the remaining part of the community or whether the more abundant microbes also respond in the same way to the disturbances. For this reason, we have analyzed the dynamics of some of the dominant groups in the water column.

#### Streamlined or *K*-Strategist Microbes

Common marine *K*-strategists showed different trends depending on the depth analyzed, although not as marked as in the picocyanobacteria group. In general, SAR11, the most abundant and widespread Alphaproteobacterial clade (Rappé et al., 2002; Giovannoni et al., 2005) and SAR86, which belongs to the class Gammaproteobacteria (Dupont et al., 2012) were found in the three depths. However, at 15 m, they decreased in the first 7 h and then increased, although the net growth, that is final/initial time, was negative (Figure 1B). Curiously, at the DCM, SAR11 clades Ia, Ib, and V increased in both bottles, while clade II and some unclassified SAR11 genomes decreased. This showed how, within the great diversity of these groups, different ecotypes could respond differently to natural perturbations in the environment. All SAR86 clades (A, E, and unclassified) exhibited a strong increase in the number of reads recruited between 7 and 14 h post-confinement (Figure 1B). These two groups have very streamlined genomes with restricted metabolism and some metabolic pathways incomplete or not present (Dupont et al., 2012; Eiler et al., 2016). They cannot use high molecular weight compounds (i.e., polysaccharides and proteins) and hence, in SAR11, they rely on a disproportionate number of ABC transporters, which are highly transcribed (Shi et al., 2011), or TonB transporters in SAR86 for the uptake of low complex nutrients. The growth of certain bacteria, like Bacteroidetes or *Alteromonas*, during the experiment can increase the availability of low molecular weight compounds by the hydrolysis of high molecular weight nutrients and promote the growth of scavenging bacteria, as previously reported in the Atlantic Ocean (Reintjes et al., 2018).

#### Archaea

Remarkably, analysis of the prokaryotic community structure at the level of phylum derived from 16S rRNA gene metagenomic fragments revealed that, during the confinement, marine archaea suffered the strongest decrease in abundance at 60 and 90 m depth (Supplementary Figure S2). MGII/III Euryarchaeota are (photo)heterotrophs that require the uptake of nutrients from the environment. They seem to be auxotrophs for certain amino acids (Haro-Moreno et al., 2017), so they compete for the available dissolved carbon and nitrogen in the form of sugars, amino acids, and small oligopeptides. Our data suggest that they perform poorly against other microbes that use similar resources. Marine Thaumarchaeota also decreased, but it was less pronounced at 60 m, and almost undetectable at 90 m. However, we do not have an explanation for this change, since they do not compete for reduced dissolved organic matter nor are inhibited by the presence of light. Our data suggest that Euryarchaeota and Thaumarchaeota phages might play a major role in their demise (see below).

### Genes Coding for Key Functions

**FIGURE 3:**
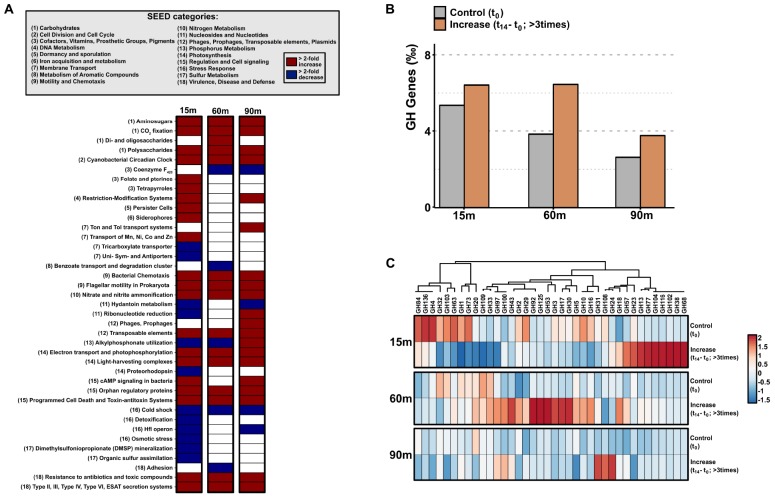
**(A)** SEED subsystems-based heatmap using the assembled coding sequences coming from contigs > 1 kb that increased in their relative abundance three times or more. Categories that increased >2-fold are indicated in red, while categories that decreased the same amount are indicated in blue. **(B)** Number of genes per 1000 genes assigned to glycoside hydrolases (GHs) detected in the t_0_ and the subset of contigs > 1 kb that increased at least three times their relative abundance, using the Carbohydrate-Active enZYmes (CAZy) database. **(C)** Heatmap of the different GH families. GH families were clustered by clade abundance.

In order to make a functional characterization of the microbial community that benefited from incubation conditions, contigs > 1 kb that increased their relative abundance three times or more after 14 h post-confinement were selected, and coding sequences were annotated against the SEED Subsystems database (Overbeek et al., 2005). Only categories that increased or decreased their abundance (>2-fold) in comparison with the initial point were selected (Figure 3A). As expected from the results obtained by genome recruitment where *Synechococcus* and *Prochlorococcus* increased at all depths, genes related to cyanobacterial activity (CO_2_ fixation, cyanobacterial circadian clock, photophosphorylation, and light-harvesting complexes) were enriched. Additionally, categories related to DNA metabolism (restriction and modification systems), motility, and chemotaxis; regulation and cell signaling; transposable elements; and virulence, disease, and defense (resistance to toxic compounds and type II/III/IV/VI secretion) systems increased at all depths. These results are in agreement with the growth of opportunistic bacteria, such as *Alteromonas*, *Limnobacter*, or *Erythrobacter*, that usually possess larger genomes and encode for these genes in their flexible genome (López-Pérez et al., 2012). We also detected changes in categories related to the uptake, synthesis, and degradation of certain compounds. For instance, we observed an enrichment in genes involved in nitrate uptake and denitrification to nitrite, and degradation of complex sugars (polysaccharides and amino sugars—chitin), at all depths; or the synthesis of certain cofactors (such as vitamin B_12_ and folates) and transport of metals (such as Mn, Ni, Co, Zn, and iron) that only increased at 15 m depth (Figure 3A). Furthermore, structural phage proteins, such as terminase and capsid increased notably at 90 m, in agreement with the changes observed in the viral community at that depth (see below). On the other hand, light-induced proteorhodopsins and genes related to the assimilation of organic sulfur and dimethylsulfoniopropionate mineralization decreased in the UP.

We also analyzed the variation in the number of genes per 1000 encoding glycoside hydrolases enzymes, involved in the degradation of complex sugars. Results showed an increase (Figure 3B) regardless of the depth analyzed, although this increment was more pronounced at the DCM. Interestingly, followed by the increase of the number of these enzymes, we noticed that glycoside hydrolases families present at the initial point were replaced with others without overlap 14 h post-confinement (Figure 3C). These patterns indicate a variation in sugars available in the environment and therefore, in the community that is capable of degrading them.

### Role of Phages in Community Structure

Viruses that infect marine microbes are an integral component of aquatic ecosystems and the most abundant entities (Suttle, 2005). They not only play an essential role in the carbon cycle but also to maintain diversity in bacterial, archaeal, and eukaryotic populations in the ocean by keeping at bay microbes with high cell densities (Corinaldesi et al., 2007; Suttle, 2007; Danovaro et al., 2008). Metagenomes (cellular fraction > 0.2 μm) contain abundant viral material due to cells retrieved while undergoing viral lysis or as temperate viruses inserted in the chromosome (López-Pérez et al., 2017). This natural amplification method increases the amount of viral DNA available that can be assembled, and several studies have been able to recover thousands of new viral contigs using this method (Mizuno et al., 2013; López-Pérez et al., 2017).

In the same way as bacterial taxa, their viruses and the phage–host dynamics may represent important players of the rare biosphere, contributing to the functional flexibility of the ecosystem. We used the same approach previously described (López-Pérez et al., 2017) to select metagenomic viral contigs from metagenomic samples and assign the host. We obtained 1089 metagenomic viral contigs longer than 10 kb for further analysis. Besides, we included 1323 viral sequences retrieved from a previous metagenomic study from the Mediterranean Sea (López-Pérez et al., 2017). To avoid redundancy, we first grouped all the sequences into clusters (>40% coverage and nucleotide sequence identity > 90%). This resulted in 1473 different viral clusters. We were able to assign putative hosts to 282 contigs (*ca*. 20% of the total) (Supplementary Table S4). The most frequent host prediction (*ca*. 63%) was Cyanobacteria, followed by Alphaproteobacteria, mainly SAR11. Unfortunately, we have not been able to detect any phage related to the *r*-strategists.

**FIGURE 4:**
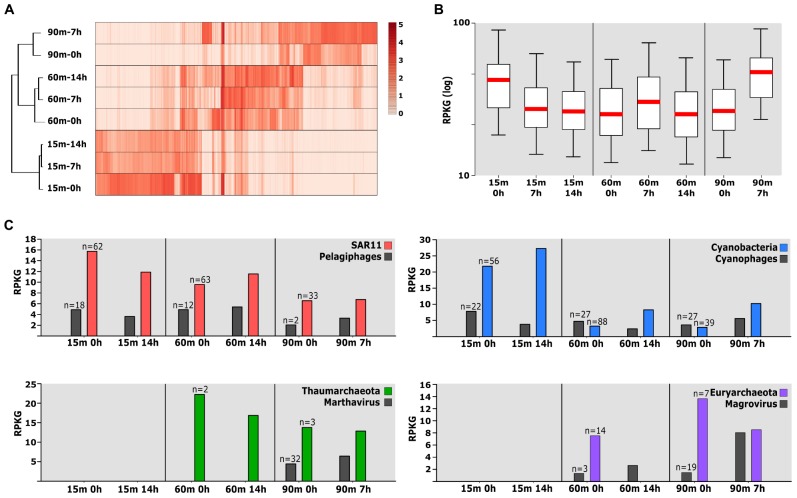
**(A)** Relative abundance of the viral cluster measured by recruitment (in RPKG) from the different metagenomes. Only those viral clusters recruiting more than 10 RPKG were taken into account. Metagenomes are clustered based on viral abundance. **(B)** Box plot representing recruitment total viral abundance per sample. **(C)** Abundance (in RPKG) of individual viral clusters and associated host populations throughout the dataset.

We have used the recruitment of metagenomic reads to elucidate possible patterns of behavior of these phages during the incubation and to analyze the role of this biotic component, which is often overlooked. We took into consideration only those clusters recruiting more than 10 RPKG of coverage with a similarity > 99%. It is remarkable that a large number of the viral clusters (*ca*. 89%) appear to be found exclusively in one single specific depth metagenome (Figure 4A). This stenobathic character is consistent with the narrow depth distributions found in the previous analysis of the prokaryotic fraction of these metagenomes (Haro-Moreno et al., 2018). Only two singletons related to pelagiphage HTVC008M were recruited in all the photic metagenomes and they could be considered eurybathic. Interestingly, unlike in bacterial recruitment where the linear representation shows all the genomic diversity of the organisms, in these viruses, we recovered reads mainly at identities higher than 99%, showing that we recovered a specific clonal lineage (Supplementary Figure S4). While at 15 m, there is a sharper decrease in the number of reads associated to viral clusters (normalized by sample), sharper in the first 7 h, the largest increase was detected at 7 h in the 90 m sample. Conversely, the viral community did not change substantially at 60 m (Figure 4B).

To refine this picture, we examined the abundance of individual viral and associated host populations throughout the dataset (Figure 4C). Although this method is limited to the number of genomes available in each sample and the host prediction, this will allow us to have a more complete view of the processes that are carried out during the incubation. While the reads associated with Cyanobacteria increased in all depths, a decrease was found in their phages in the UP and DCM regions (Figure 4C). Mixotrophy can provide a competitive advantage for the bacteria in darkness while the infection by cyanophages seems to be very conditioned by the presence of light. Similar results have been obtained in experiments on cyanophage infection under daily light–dark (diel) cycles suggesting that the adsorption of some cyanophages to their host cells is light-dependent (Ni and Zeng, 2016). However, in deeper waters where light is scarce (90 m), the trend changes, and we observed a slight growth of both (bacteria and phage). On the other hand, the temporal pattern of pelagiphages abundance appeared tightly coordinated with that of SAR11 (Figure 4C).

Archaea are ubiquitous and abundant in marine ecosystems; however, little is known about the interaction between virus and archaea occurring in marine environments (Danovaro et al., 2016; Roux et al., 2016). In fact, the first viruses that infect Marine Group I Thaumarchaeota and Marine group II Euryarchaeota have recently been discovered by metagenomics (Philosof et al., 2017; López-Pérez et al., 2018). The relative abundance of Euryarchaeota in the DCM sample was higher at the beginning, disappearing entirely after 14 h (see above). However, the abundance of their only known viruses (magrovirus) slightly increases after 14 h (Figure 4C). In deeper waters (90 m), which is the usual habitat of these microbes, there was a strong negative correlation. The same correlation, although less pronounced, could be observed for the other group of Archaea at 90 m (Figure 4C).

The reasons for such rapid changes are obscure, but we would like to advance a hypothesis that we call “wall-chain-reaction.” While the pelagic assemblage drifts along with the water mass in the ocean, the ratio virus/host is kept relatively constant. Confinement will immediately change the situation, particularly near the walls of the container where the stochastic movement of cells and viruses bouncing off the walls will result in a higher local concentration of both. This could change the delicate predator–prey balance favoring lytic viruses that could lead to a chain reaction starting near the wall that would result in massive lysis of some preys. This could be the case of the Euryarchaea which populations were shown to collapse rapidly. Cell lysis would lead to a massive release of organic carbon that would be first taken up by *r*-strategists like *Erythrobacter* or *Alteromonas*. This is the first time where metagenomic assembly and recruitment have been applied to analyze phage–host dynamics and viral diversity in response to the incubations in marine samples. Phages of these low-abundance taxa that control their populations after environmental disturbances could be considered as the “rare virosphere” (Holmfeldt et al., 2013). This situation is similar to the evolutionary model of equilibrium where host-specific viruses control the bacterial population proposed as “constant-diversity” dynamics (Rodriguez-Valera et al., 2009).

## Conclusion

As far as we know, this is the first time where metagenomic assembly and recruitment have been applied to analyze the microbial community response to the “bottle effect” in marine samples. Although the change in the overall community structure is not dramatic at the level of the major groups, we found clear ecotype succession, suggesting high intra-species diversity that provides different adaptive mechanisms at the level of population rather than at individual level. It seems clear that some well-known *r*-strategists that can grow rapidly increase during incubation, while in the sample processed directly (*t*_0_), they are probably only in minute amounts that are not ecologically relevant. Furthermore, we were able to analyze the phage–host dynamic through the assembly and recruitment of viral genomes that are retrieved while undergoing the lytic cycle (López-Pérez et al., 2017), showing the high impact of this biotic factor in deeper waters. The “bottle effect” might act by increasing the probability of contact between viruses and prokaryotic cells resulting to be one of the main determinants of community changes. Our results highlight the importance of processing the samples immediately after their collection to obtain a reliable representation of the *in situ* prokaryotic community.

## Data Availability Statement

The datasets generated for this study can be found in the NCBI SRA and are available under BioProject accession numbers PRJNA352798 (Med-OCT2015-15m [SRR5007106], Med-OCT2015-60m [SRR5007118], Med-OCT2015-90m [SRR5007139], Med-OCT2015-15m7h [SAMN10839273], Med-OCT2015-15m14h [SAMN10839292], Med-OCT2015-60m7h [SAMN10839294], Med-OCT2015-60m14h [SAMN10839296], and Med-OCT2015-90m7h [SAMN10839330]). The reconstructed genomes have been deposited as BioSample SAMN10841217 to SAMN10841310 under BioProject PRJNA352798.

## Author Contributions

ML-P conceived the study. FR-V helped with the analysis and to writing the manuscript. JH-M analyzed the data together with ML-P and contributed to the writing of the manuscript.

## Conflict of Interest

The authors declare that the research was conducted in the absence of any commercial or financial relationships that could be construed as a potential conflict of interest.
