# Supplementary material for: Prokaryotic Population Dynamics and Viral Predation in a Marine Succession Experiment Using Metagenomics
Source: Front Microbiol. 2019 Dec 19;10:2926. doi: 10.3389/fmicb.2019.02926 (PMC6931264; doi:10.3389/fmicb.2019.02926)
Supplement: Supplementary file 1 [file Data_Sheet_1.PDF]

Prokaryotic population dynamics and viral predation in a marine succession experiment using metagenomics

Jose M. Haro-Moreno[1], Francisco Rodriguez-Valera[1,2], Mario López-Pérez[1*]

[1]Evolutionary Genomics Group, División de Microbiología, Universidad Miguel Hernández, Apartado 18, San Juan 03550, Alicante, Spain. [2]Laboratory for Theoretical and Computer Research on Biological Macromolecules and Genomes, Moscow Institute of Physics and Technology, Moscow, Russia.

[*]Corresponding autor: mario.lopezp@umh.es

Evolutionary Genomics Group, División de Microbiología, Universidad Miguel Hernández, Apartado 18, San Juan de Alicante, 03550 Alicante, Spain.

Phone +34-965919313, Fax +34-965 919457

SUPPLEMENTARY INFORMATION

Supporting information Fig S1. Cladogram representing a Bray-Curtis dissimilarity (%) among the 16S rRNA gene fragments extracted from metagenomic samples, collected during consecutive years and seasons (summer – stratified water column, winter – mixed water column). In blue, metagenomes analysed in this study. The stratified water column is divided into three layers, Upper Photic, Deep Chlorophyll Maximum and Lower Photic.

Supporting information Fig S2. Population dynamics based on 16S rRNA gene fragments (raw reads) at phylum-level. The phylum Proteobacteria was divided into its class-level classification. Groups with abundance values lower than 1% in any of the metagenomes were discarded. Line of the circles of the samples from this study is highlighted in red.

Supporting information Fig S3. Heatmap showing the recruitment values of 95 genomes that recruited at least 3 RPKG in any of the 33 metagenomes used, collected from several locations, years and seasons in the Mediterranean Sea. In blue, metagenomes analysed in this study.

Supporting information Fig S4. Fragment recruitment plots of putative viral genomes over time in the three depths tested.

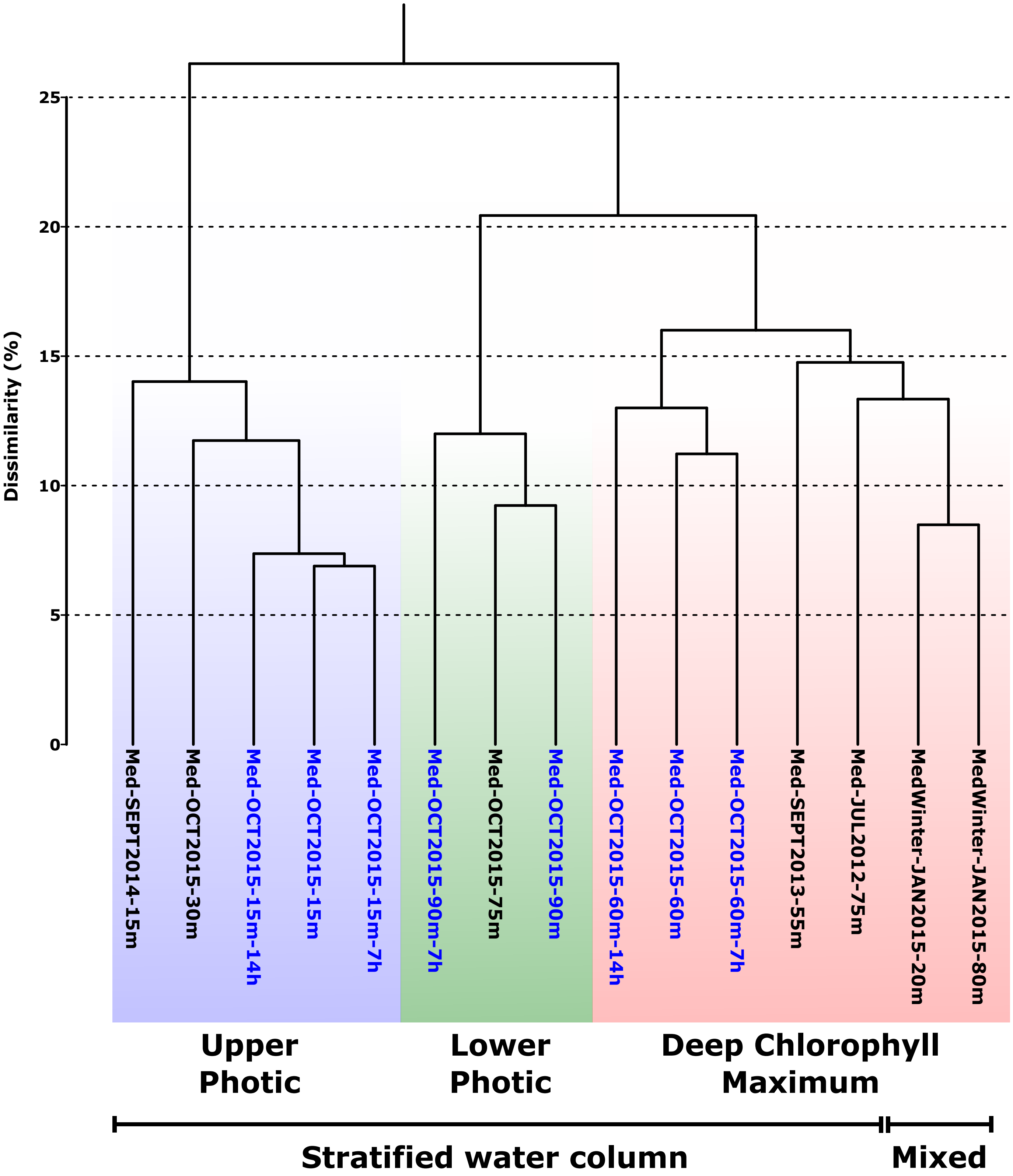

Dissimilarity (%)
25
20
15
10
5
0
Med-SEPT2014-15m
Med-OCT2015-30m
Med-OCT2015-15m-14h
Med-OCT2015-15m
Med-OCT2015-15m-7h
Med-OCT2015-90m-7h
Med-OCT2015-75m
Med-OCT2015-90m
Med-OCT2015-60m-14h
Med-OCT2015-60m
Med-OCT2015-60m-7h
Med-SEPT2013-55m
Med-JUL2012-75m
MedWinter-JAN2015-20m
MedWinter-JAN2015-80m
Upper Photic
Lower Photic
Deep Chlorophyll Maximum
Stratified water column
Mixed

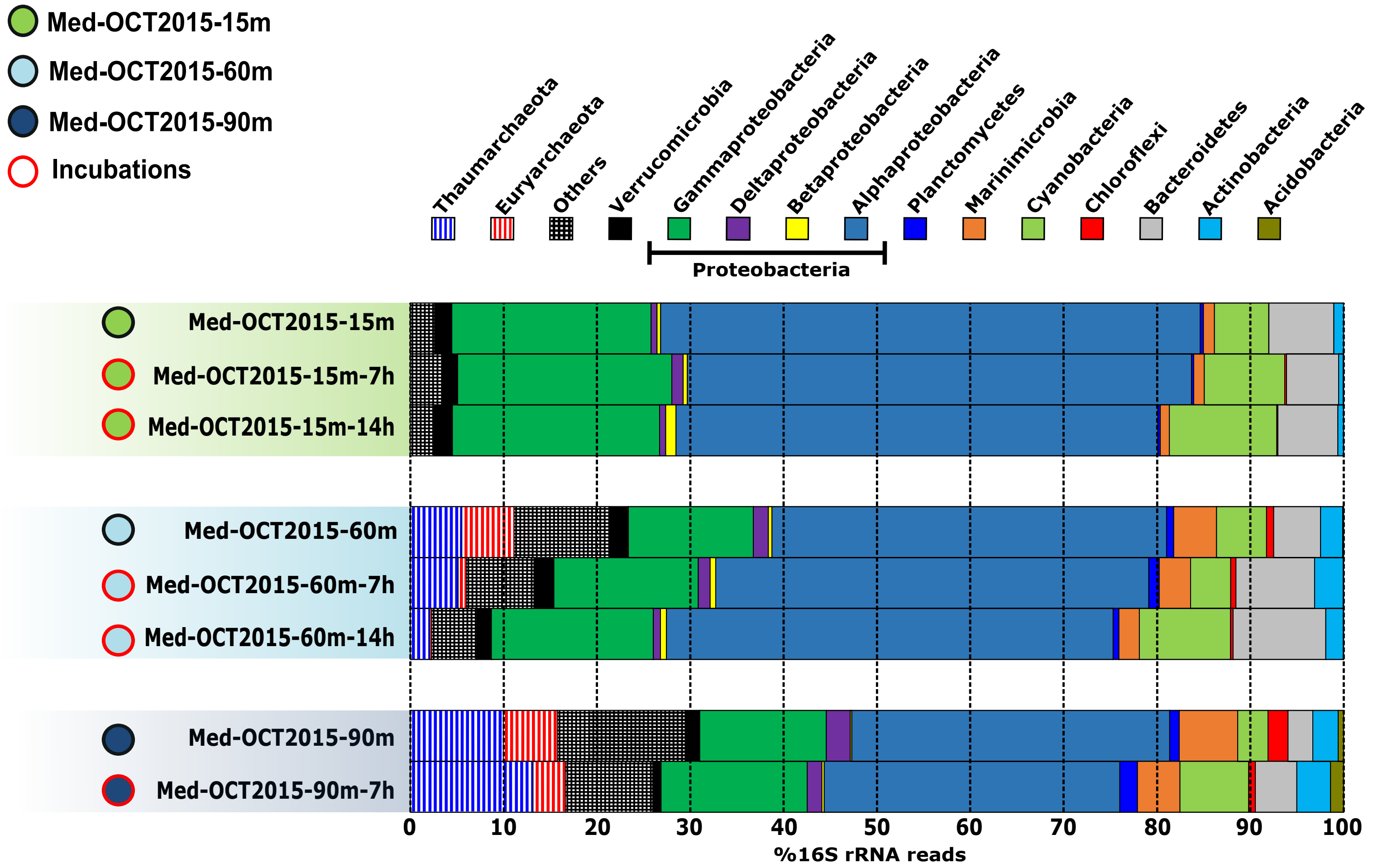
Med-OCT2015-15m
Med-OCT2015-60m
Med-OCT2015-90m
Incubations
Thaumarchaeota
Euryarchaeota
Others
Verrucomicrobia
Gammaproteobacteria
Deltaproteobacteria
Betaproteobacteria
Alphaproteobacteria
Planctomycetes
Marinimicrobia
Cyanobacteria
Chloroflexi
Bacteroidetes
Actinobacteria
Acidobacteria
Proteobacteria
Med-OCT2015-15m
Med-OCT2015-15m-7h
Med-OCT2015-15m-14h
Med-OCT2015-60m
Med-OCT2015-60m-7h
Med-OCT2015-60m-14h
Med-OCT2015-90m
Med-OCT2015-90m-7h
0
10
20
30
40
50
60
70
80
90
100
%16S rRNA reads

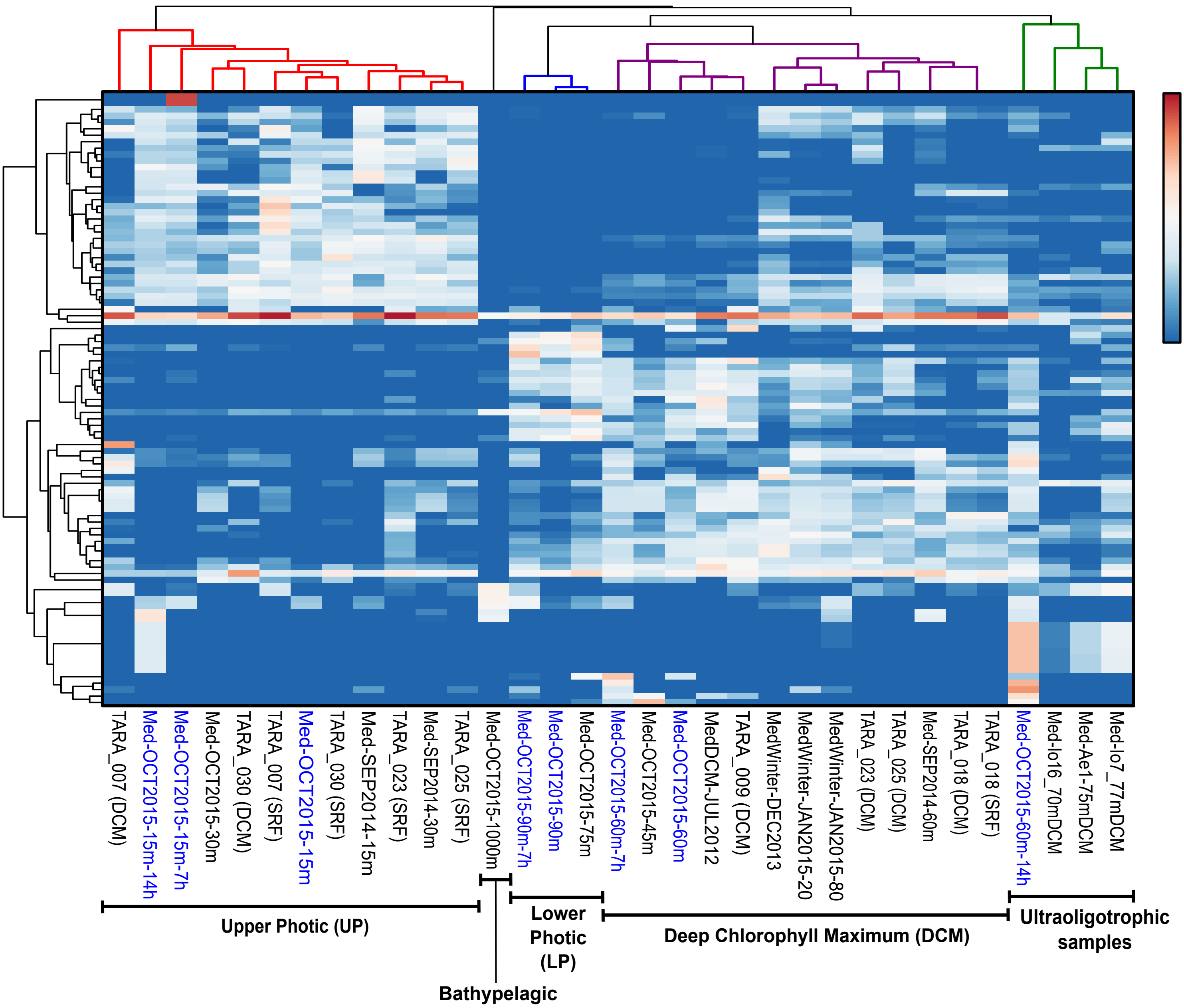

TARA_007 (DCM)
Med-OCT2015-15m-14h
Med-OCT2015-15m-7h
Med-OCT2015-30m
TARA_030 (DCM)
TARA_007 (SRF)
Med-OCT2015-15m
TARA_030 (SRF)
Med-SEP2014-15m
TARA_023 (SRF)
Med-SEP2014-30m
TARA_025 (SRF)
Med-OCT2015-1000m
Med-OCT2015-90m-7h
Med-OCT2015-90m
Med-OCT2015-75m
Med-OCT2015-60m-7h
Med-OCT2015-45m
Med-OCT2015-60m
MedDCM-JUL2012
TARA_009 (DCM)
MedWinter-DEC2013
MedWinter-JAN2015-20
MedWinter-JAN2015-80
TARA_023 (DCM)
TARA_025 (DCM)
Med-SEP2014-60m
TARA_018 (DCM)
TARA_018 (SRF)
Med-OCT2015-60m-14h
Med-Io16_70mDCM
Med-Ae1-75mDCM
Med-Io7_77mDCM
Upper Photic (UP)
Bathypelagic
Lower Photic (LP)
Deep Chlorophyll Maximum (DCM)
Ultraoligotrophic samples
6
5
4
3
2
1
0

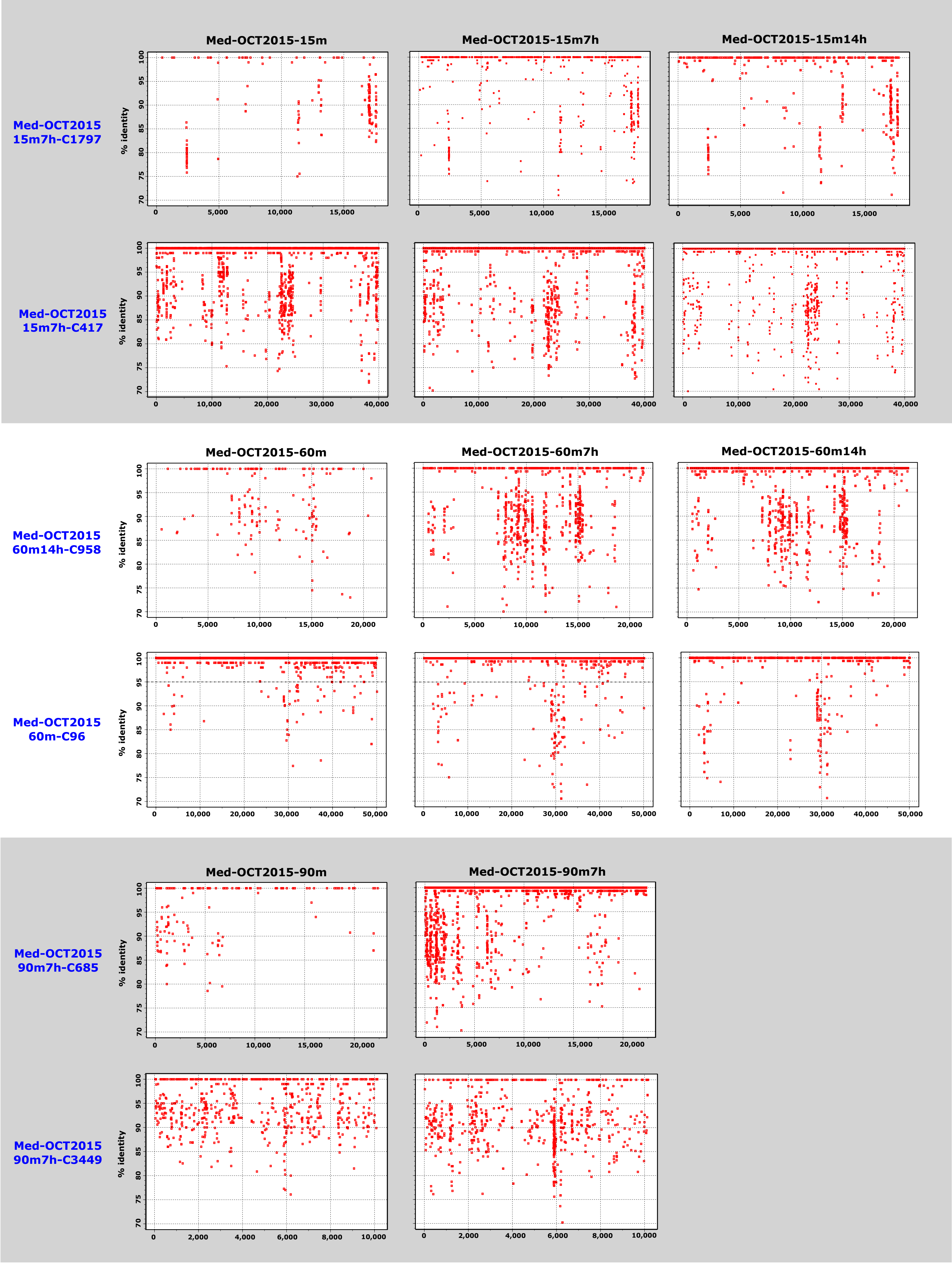

Med-OCT2015-15m
Med-OCT2015-15m7h
Med-OCT2015-15m14h
Med-OCT2015 15m7h-C1797
Med-OCT2015 15m7h-C417
Med-OCT2015-60m
Med-OCT2015-60m7h
Med-OCT2015-60m14h
Med-OCT2015 60m14h-C958
Med-OCT2015 60m-C96
Med-OCT2015-90m
Med-OCT2015-90m7h
Med-OCT2015 90m7h-C685
Med-OCT2015 90m7h-C3449
% identity
100
95
90
85
80
75
70
0
5,000
10,000
15,000
20,000
30,000
40,000
50,000
2,000
4,000
6,000
8,000
